# Toward a translational molecular ratchet: face-selective translation coincident with deuteration in a pseudo-rotaxane

**DOI:** 10.1038/s41598-018-27226-2

**Published:** 2018-06-12

**Authors:** Akihito Hashidzume, Akihiro Kuse, Tomoya Oshikiri, Seiji Adachi, Mitsutaka Okumura, Hiroyasu Yamaguchi, Akira Harada

**Affiliations:** 1 Graduate School of Science, Osaka University, 1-1 Machikaneyama-cho, Toyonaka, Osaka, 560-0043 Japan; 2 Research Institute for Electronic Science, Hokkaido University, Kita 21-jo, Nishi 10-chome, Kita-ku, Sapporo, 001-0021 Japan; 3 JST-ImPACT, Chiyoda-ku, Tokyo, 100-8914 Japan

## Abstract

In the molecular world, molecular ratchets can realize the unidirectional movement in molecular machines. However, construction of artificial molecular ratchets has been still a great challenge. In this study, we investigate the formation of pseudo-rotaxane of a newly designed two-station axis molecule with α-cyclodextrin (α-CD) and the deuteration of acidic protons in the axis in D_2_O by ^1^H NMR at varying temperatures. Using the NMR data, we roughly estimate apparent rate constants for association, dissociation, and translation of α-CD during the pseudo-rotaxane formation based on a simplified kinetic model. These rate constants are indicative of face-selective and ratchet-like translation of α-CD on the axis because of the 2-methylpyridinium residues in the axis. We also evaluate apparent first-order rate constants for the deuteration. Comparison of these rate constants indicates that the face-selective translation of α-CD somehow couples with the deuteration. On the basis of this study, it is concluded that a translational molecular ratchet can be constructed using a large energy gradient with appropriate energy barriers and an enthalpically-driven coupled reaction.

## Introduction

Living organisms utilize translational and rotational molecular motors, e.g., kinesin, dynein, and ATP synthase, when they have to move or operate^1,2^. These biological molecular motors are based on Brownian ratchet, which withdraws the direction from thermal fluctuations on a sawtooth-shape potential surface^3–7^, or on coupling with a chemical reaction driven enthalpically^1,2^. In the real world, the ratchet mechanism regulates the direction of movement. Similarly, in the molecular world, molecular ratchets can realize the unidirectional movement in molecular machines^8–11^. However, construction of artificial molecular ratchets has been still a great challenge. Some groups have published pioneering works on artificial molecular ratchets. Kelly *et al*.^12–14^ devoted their efforts toward rotational molecular ratchets using triptycene and pawl moieties. Leigh *et al*.^15^, Arduini *et al*.^16–19^, and Chen *et al*.^20^ have reported translational molecular ratchet based on a rotaxane or pseudo-rotaxane, in which the direction of rotor component is regulated by attaching and detaching a pawl moiety on the axis component. Stoddart *et al*.^21^ have elaborated an artificial molecular pump based on a pseudo-rotaxane of cyclobis(paraquat-*p*-phenylene) and an axis molecule with an isopropyl group as energy barrier, in which redox and thermal stimuli cause unidirectional translation of the rotor component. Leigh *et al*.^22–24^ have realized a molecular information ratchet based on a rotaxane possessing a stilbene moiety, of which the *Z*–*E* ratio in the photostationary state depends on the photosensitizer. We have reported face-selective translation of the rotor, i.e., α-cyclodextrin (α-CD), in pseudo-rotaxanes composed of decamethylene stations and methyl-substituted pyridinium residues^25–28^, in which the 2-methyl group on pyridinium acts like a barb of fish hook for α-CD. We have also investigated deuteration of acidic protons in a two-station axis molecule catalyzed by α-CD in deuterium oxide (D_2_O) during the formation of pseudo-rotaxane^29^. Here we show a pseudo-rotaxane that realizes the face-selective translation of α-CD coincident with the α-CD-catalyzed deuteration in D_2_O, aiming at a translational molecular ratchet.

## Results

### The formation of pseudo-rotaxanes from the two-station axis molecule and α-cyclodextrin

**Figure 1:**
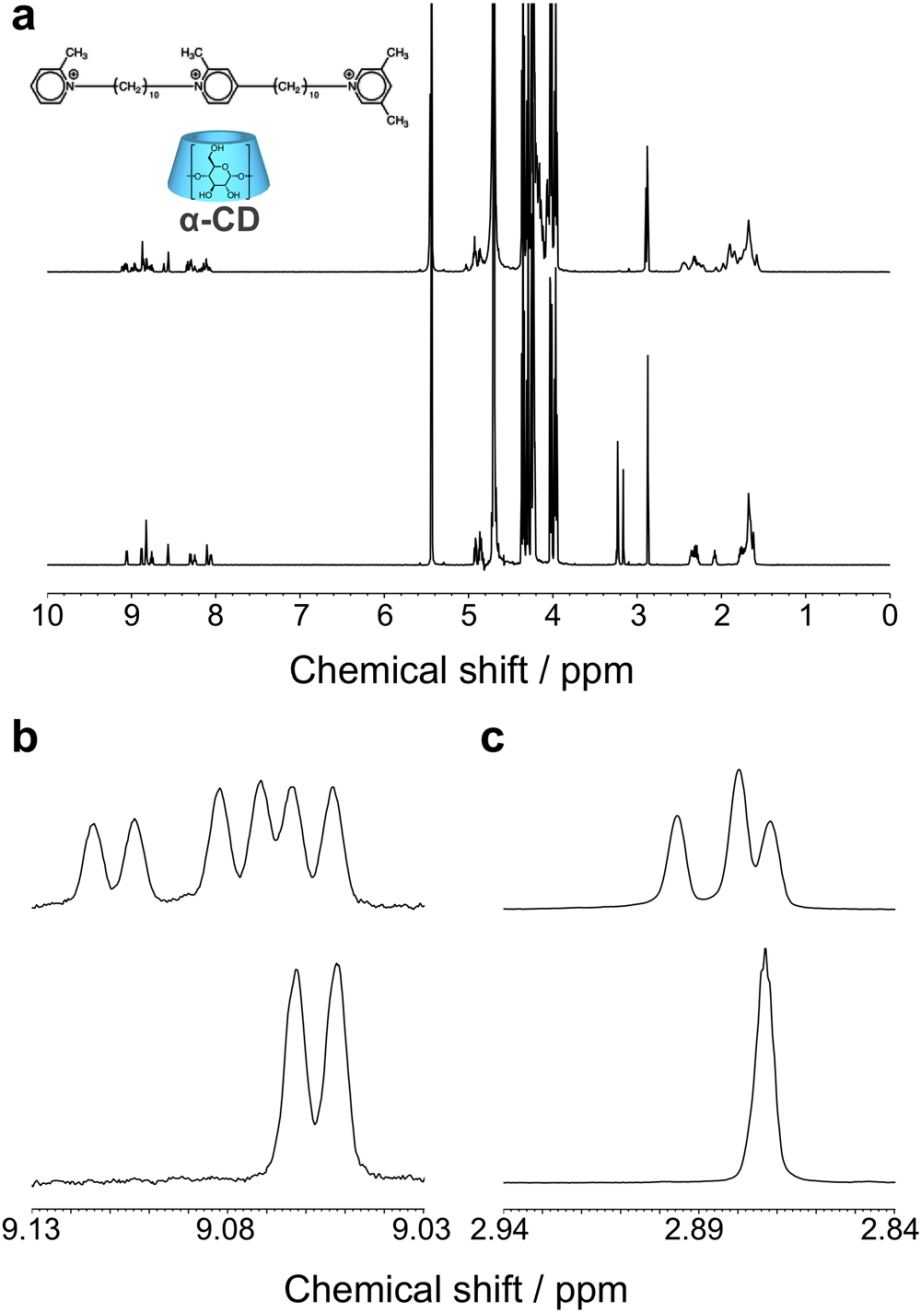
^1^H NMR spectra for a binary mixture containing the two-station axis molecule (2.3 mmol kg^−1^) and α-CD (9.1 mmol kg^−1^) in D_2_O at 70 °C just after heating (lower) and after heating for 7 days (upper). (**a**) Whole spectra. (**b**) and (**c**) Partial spectra.

The two-station axis molecule used in this study bears a 3,5-dimethylpyridinium residue, as a stopper for α-CD, on one end, and a 2-methylpyridinium residue, as a regulator for the direction of α-CD, on the other end. The two stations are connected with another 2-methylpyridinium residue. These two 2-methylpyridinium residues act like barbs. The two-station axis molecule was prepared step by step in a manner similar to our previous reports (see Supplementary information)^28,29^. Figure 1 shows ^1^H NMR spectra of a binary mixture of the two-station axis molecule and α-CD measured in D_2_O at 70 °C. The lower spectrum of Fig. 1a exhibits a simple superimposition of the signals of the two-station axis molecule and α-CD just after heating at 70 °C, whereas the upper spectrum shows new separate signals ascribable to the two-station axis molecule included by α-CD as well as those for the free axis molecule after 7 days at 70 °C. This is because the exchange between free and complexed states is slower than the time scale of NMR due to the positive charge and bulkiness of the 2-methylpyridium residues. The proton at 6-position of the 2-methylpyridinium gate and the methyl protons of the 3,5-dimethylpyridinium stopper indicated well resolved signals ascribed to the free and two complexed ones, as can be seen in Fig. 1b,c, respectively. On the basis of several two-dimensional NMR data and our previous results^25^, signals at 9.11 and 9.08 ppm are ascribed to the first station included by α-CD from the primary and secondary hydroxy sides, respectively, and signals at 2.90 and 2.88 ppm are assigned to the second station included by α-CD from the primary and secondary hydroxy sides, respectively (Fig. S2 in Supplementary information).

**Figure 2:**
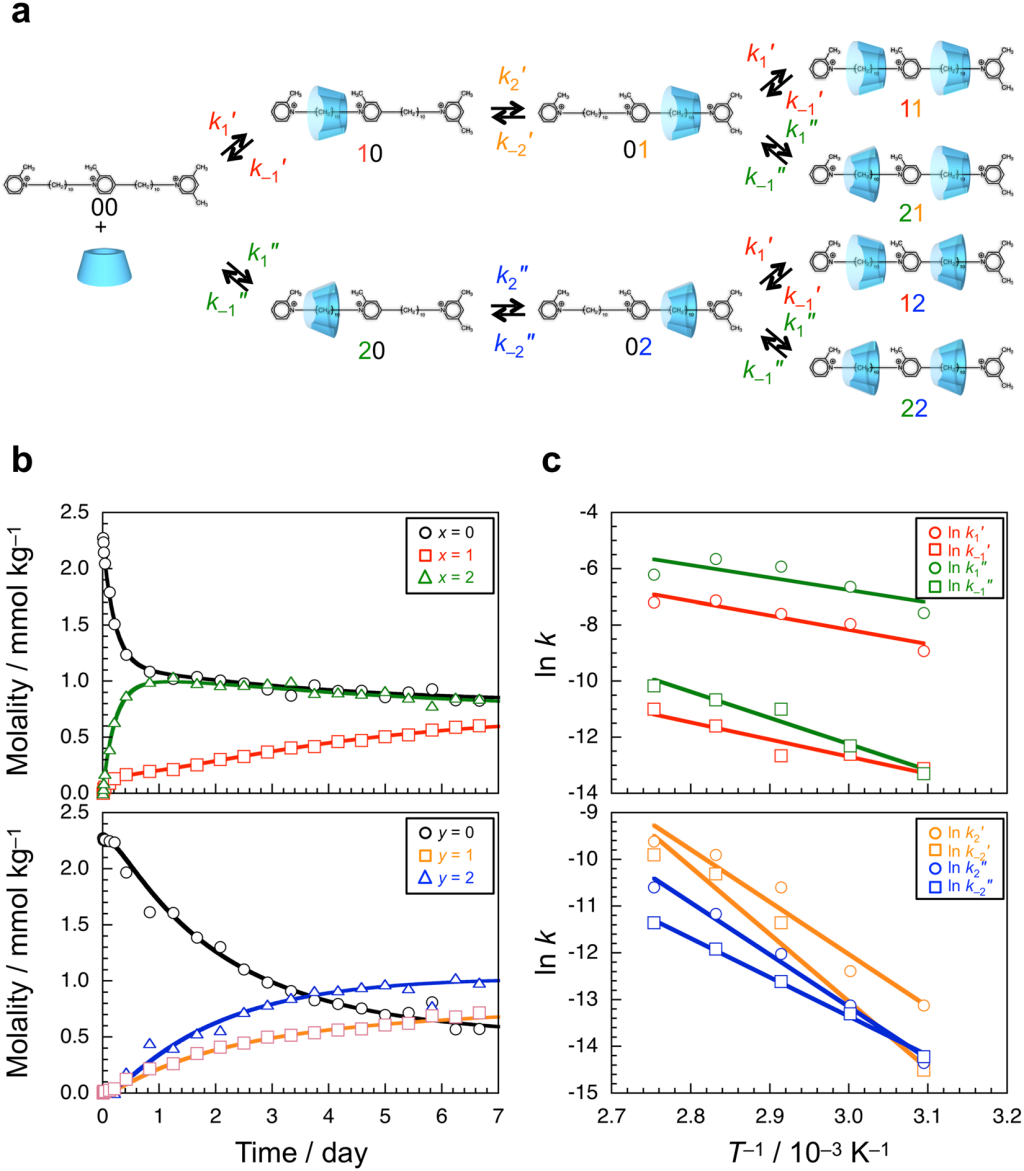
Kinetic analysis of the formation of pseudo-rotaxane from the two-station axis molecule and α-CD. (**a**) A simplified kinetic model. (**b**) Time evolution of the concentrations of free and complexed stations of the two-station axis molecule in D_2_O at 70 °C; the first (upper) and second stations (lower), and free (circle) and included by α-CD from the primary (square) and secondary sides (triangle), respectively. (**c**) Arrhenius plots of the rate constants estimated.

Using the spectra measured at 70 °C and different times, the molalities of free and two complexed first and second stations were determined by deconvolution, and were plotted in Fig. 2b against time. As shown in the upper figure of Fig. 2b, the molality of free first station rapidly decreases within a day, and levels off at ca. 1.0 mmol kg^−1^ (black circles). The molality of first station included by α-CD from the secondary hydroxy side increases rapidly corresponding to the rapid decrease in the molality of free first station, reaches a maximum at ca. 1 day, and then decreases gradually (green triangles). On the other hand, the molality of first station included by α-CD from the primary hydroxy side increases gradually to ca. 0.7 mmol kg^−1^ over 7 days (red squares). As can be seen in the lower figure of Fig. 2b, the molality of free second station decreases more slowly than does that of free first station (black circles). As the molality of free second station decreases, the molalities of second station included by α-CD from both the primary and secondary hydroxy sides increase (orange squares and blue triangles, respectively). The molality of second station included by α-CD from the secondary hydroxy side is higher than that included from the primary hydroxy side, corresponding to those of the first station. The same experiments were also conducted at different temperatures, i.e., 50, 60, 80, and 90 °C (Fig. S3 in Supplementary information).

Here we propose a simplified kinetic model to analyze the kinetics for the formation of pseudo-rotaxane (Fig. 2a). The state of two-station axis molecule is defined as *xy*, where *x* and *y* denote the inclusion states of the first and second stations, respectively. Values of *x* and *y* are 0, 1, or 2; 0 means the free state, and 1 and 2 denote the complexed states, in which the station is included by α-CD from the primary and secondary hydroxy sites, respectively. The two-station axis molecule can thus take one of nine states, i.e., 00, 10, 20, 01, 02, 11, 21, 12, or 22. The association and dissociation rate constants between the 00 and 10 states are defined as *k*_1_′ and *k*_−1_′, respectively. Similarly, the association and dissociation rate constants between the 00 and 20 states are defined as *k*_1_″ and *k*_−1_″, respectively. The rate constants for forward and backward reactions between 10 and 01 are defined as *k*_2_′ and *k*_−2_′, and those between 20 and 02 are defined as *k*_2_″ and *k*_−2_″, respectively. For simplicity, it is assumed that the association and dissociation rate constants for the first station are independent of the state of second station. As described in Supplementary information, we roughly estimated the eight rate constants by fitting the calculated molalities, i.e., [0 *y*] (=[00] + [01] + [02]), [1 *y*] (=[10] + [11] + [12]), [2 *y*] (=[20] + [21] + [22]), [*x*0] (=[00] + [10] + [20]), [*x*1] (=[01] + [11] + [21]), and [*x*2] (=[02] + [12] + [22]), to the experimental data in Figs 2b and S3 in Supplementary information, where [*xy*] is the molality of *xy* state. As can be seen in these figures, the calculated molalities are in fairly good agreement with the experimental data using an appropriate set of values for the rate constants. It should be noted that it was almost impossible to determine sets of the rate constants unambiguously, but it was feasible to obtain the magnitude relationship of the rate constants at each temperature. Table S1 in Supplementary information summarizes the rate constants. In the whole temperature range examined, *k*_1_″ > *k*_1_′, *k*_−1_″ ≥ *k*_−1_′, *k*_2_′ > *k*_2_″, and *k*_−2_′ ≥ *k*_−2_″. This may be because the numbers of α-D-glucopyranose units of unstable conformation in α-CD are different upon passing the 2-methylpyridinium units from its primary and secondary hydroxy sides^30^. It is noteworthy that *k*_2_′ > *k*_−2_′ and *k*_2_″ > *k*_−2_″, indicating that α-CD moves preferably from the first to second stations presumably because of the thermodynamic stability, which may be ratchet-like movement. From the intercepts and slopes in the Arrhenius plot (Fig. 2c), apparent frequency factors (*A*_app_) and activation energies (*E*_app_) were roughly estimated although we would not discuss these values in detail (Table S2 in Supplementary information).

### α-Cyclodextrin-catalyzed deuteration of the two-station axis molecule

**Figure 3:**
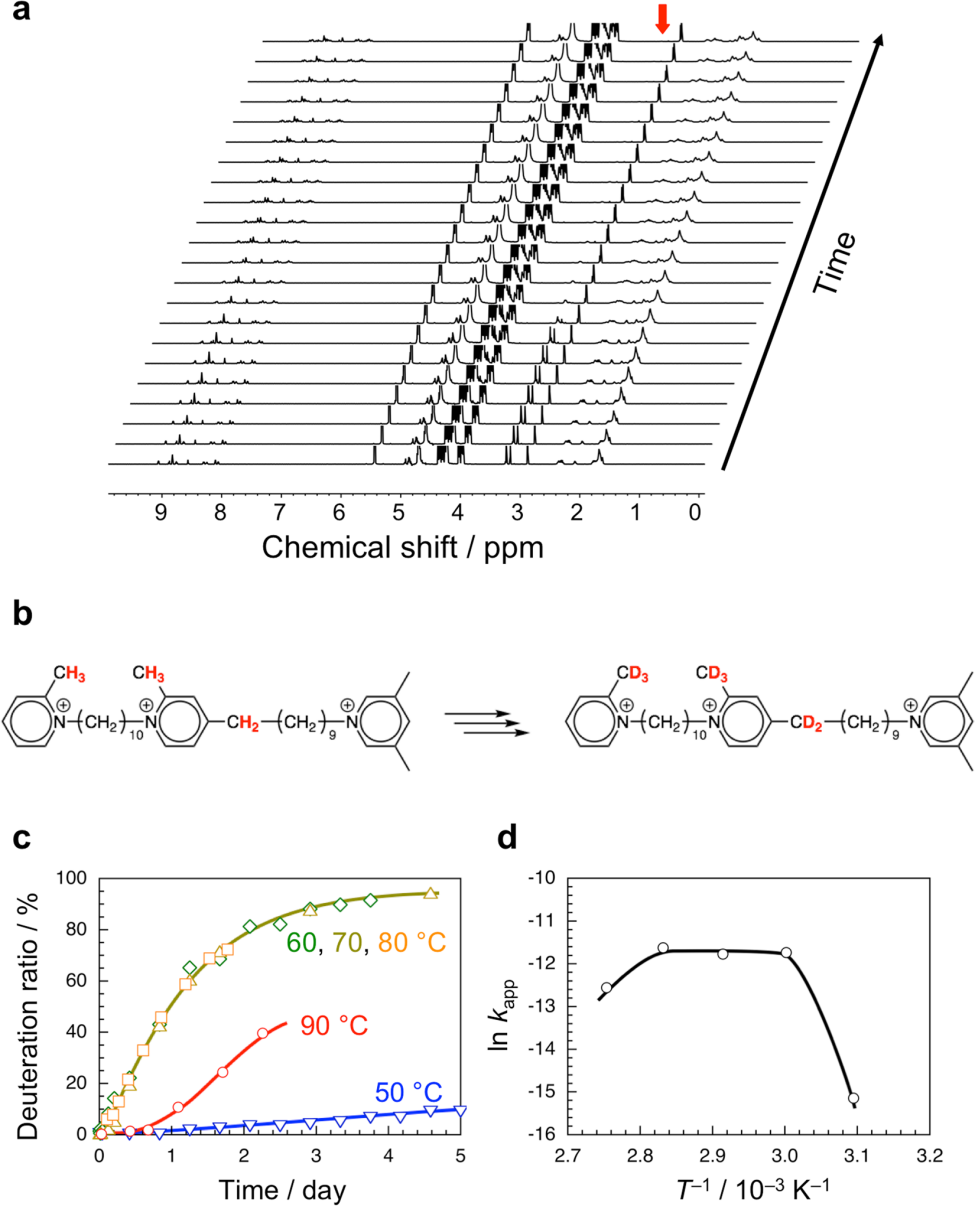
Deuteration of the two-station axis molecule in D_2_O catalyzed by α-CD. (**a**) Time evolution of ^1^H NMR spectra for a binary mixture of the two-station axis molecule (2.3 mmol kg^−1^) and α-CD (9.1 mmol kg^−1^) in D_2_O at 70 °C. (**b**) Scheme of the deuteration of the two-station axis molecule. (**c**) Deuteration ratio as a function of time for the two-station axis molecule in D_2_O in the presence of α-CD at different temperatures. (**d**) Arrhenius plot of the apparent rate constant for deuteration of the two-station axis molecule in D_2_O in the presence of α-CD.

During the formation of pseudo-rotaxane, we observed a considerable reduction in the intensity of signals in the region of 3.2–3.4 ppm assigned to protons in the methyl and methylene groups on the 2- and 4-positions of pyridinium, as shown in Fig. 3a^29^. It is known that protons in the methyl groups on the 2- and 4-positions of pyridinium are acidic because of the resonance effect^31^. In D_2_O, the acidic protons may be replaced with deuterons D^+^ of D_2_O, leading to the dissappearance of ^1^H NMR signals. To confirm the deuteration, the two-station axis molecule was measured by mass spectroscopy (MS) before and after heating in D_2_O at 70 °C in the presence of α-CD (Fig. S4 in Supplementary information). As can be seen in Fig. S4a in Supplementary information, the MS chart for the axis molecule just dissolved in H_2_O at room temperature contains the trivalent molecular ion peak at *m*/*z* = 190.83 (i.e., *m* = 572.49). On the other hand, the MS chart for the axis molecule heated in D_2_O at 70 °C for 2 days in the presence of α-CD exhibits a series of signals centered at *m*/*z* = 192.84, which is assignable to a trivalent molecular ion peak of *m* = 578.52 (Fig. S4b in Supplementary information). These NMR and MS data confirm the deuteration of the axis molecule after heating in D_2_O at 70 °C in the presence of α-CD (Fig. 3b).

**Figure 4:**
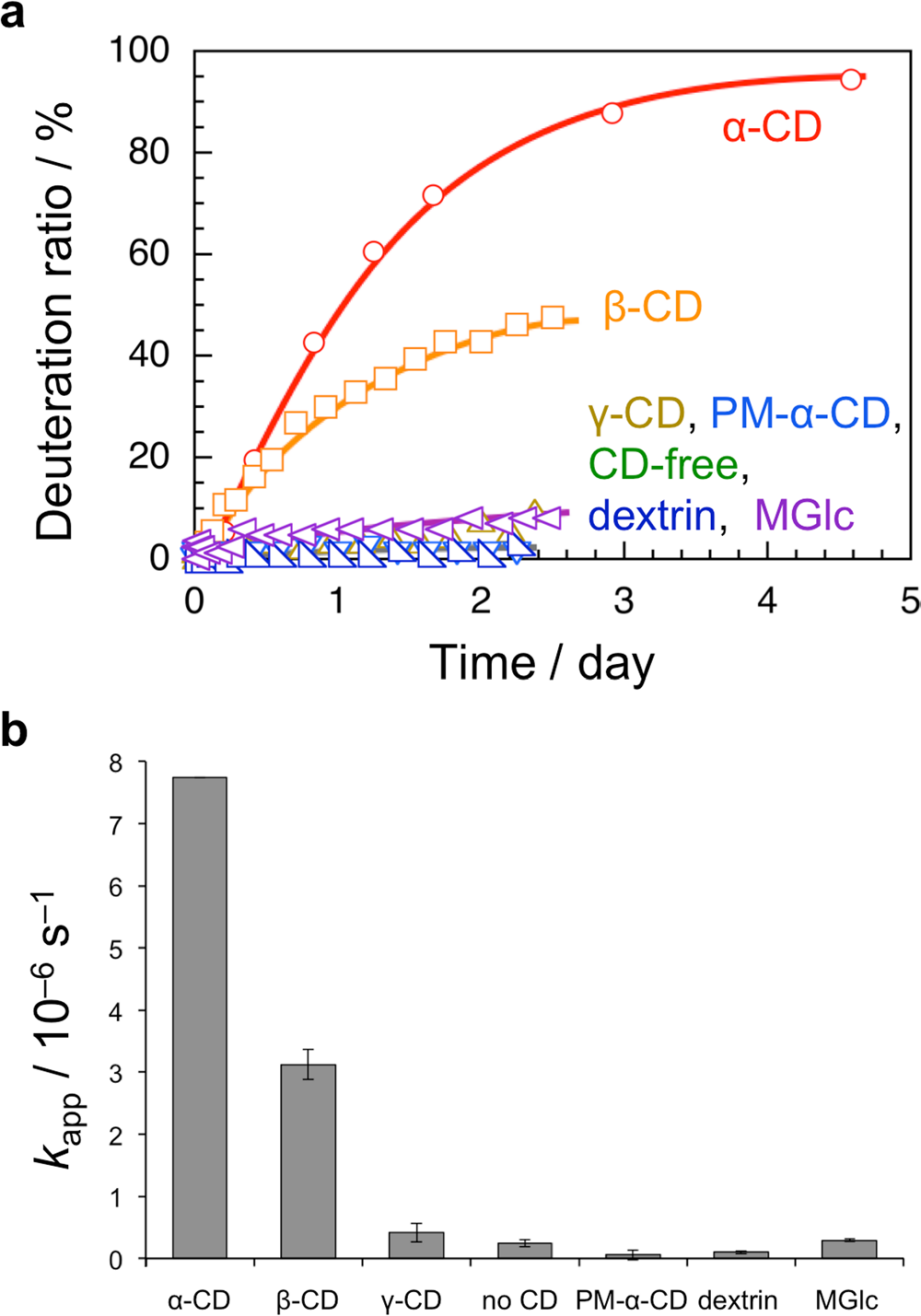
Deuteration of the two-station axis molecule in D_2_O in the absence and presence of CDs and saccharides. (**a**) Deuteration ratio as a function of time for the two-station axis molecule in D_2_O at 70 °C. (**b**) Apparent rate constants *k*_app_ for the deuteration of the two-station axis molecule in D_2_O at 70 °C in the absence and presence of α-CD, β-CD, γ-CD, PM-α-CD, dextrin, and MGlc.

To study the effect of α-CD on the deuteration, deuteration experiments of the two-station axis molecule were also carried out in D_2_O at 70 °C in the absence and presence of other CDs or saccharides, i.e., β-CD, γ-CD, permethylated α-CD (PM-α-CD), dextrin, and methyl α-D-glucopyranoside (MGlc). The deuteration ratios were quantified using the ratio of area intensities of signals due to the eight protons of methyl and methylene to that of the phenyl proton on the 4-position of the 3,5-dimethylpyridinium residue, which did not undergo deuteration. The deuteration ratios were then plotted in Fig. 4a against time for all the systems examined. In the presence of α-CD, the deuteration ratio markedly increases with time and becomes almost quantitative after 4.5 days. In the presence of β-CD, the deuteration ratio also increases significantly and reaches 45% after 2.5 days. On the other hand, in the absence of CD, or in the presence of γ-CD, PM-α-CD, dextrin, or MGlc, the deuteration ratio does not increase significantly. Given that the deuteration is pseudo-first-order, apparent first-order-rate constants (*k*_app_) were determined from slopes of the first-order-plots. Figure 4b and Table S3 in Supplementary information compare the *k*_app_ values. The deuteration reactions in the presence of α-CD and β-CD exhibit higher *k*_app_ ((7.7 ± 0.2) × 10^−6^ and (3.1 ± 0.1) × 10^−6^ s^−1^ for α-CD and β-CD, respectively). On the other hand, *k*_app_ values were much smaller under other conditions (~10^−7^ s^−1^). ^1^H NMR spectra indicated that α-CD and β-CD included the axis molecule to yield a rather stable pseudo-rotaxane whereas the others did not. It is thus concluded that pseudo-rotaxane structure is critical for the deuteration of two-station axis molecule.

The α-CD-catalyzed deuteration of the two-station axis molecule in D_2_O was examined at different temperatures. Figure 3c compares the deuteration ratios as a function of time at 50, 60, 70, 80, and 90 °C. At 50 °C, the deuteration does not occur significantly. At 60, 70, and 80 °C, the increases in deuteration ratio are almost the same. At 90 °C, however, the deuteration ratio slowly increases after an induction period of ca. 0.7 day and reaches ca. 40% after 2.4 days. Values of *k*_app_ were evaluated from the slopes of first-order plots at different temperatures. (The *k*_app_ value at 90 °C was evaluated based on the first-order plots after the induction period.) Table S4 in Supplementary information summarizes the *k*_app_ values estimated. The Arrhenius plot does not obey a single straight line (Fig. 3d); the slope is almost 0 in the region of 60–80 °C and negative at higher temperatures, 80–90 °C. This may be because the deuteration of the axis molecule catalyzed with α-CD in D_2_O proceeds via a certain metastable state, which is not stable enough at temperatures as high as 90 °C especially in an earlier stage. The formation of metastable states has been supported by density functional theory (DFT) calculations for model systems, as can be seen in Fig. S5 in Supplementary information. It should be noted here that *k*_app_ may be underestimated because once deuteration occurs exchange of deuterium between a D atom on the methyl or methylene and D in D_2_O becomes non-detectable. It is important which hydroxy side of α-CD is more effective for the deuteration. We looked closely at the signals ascribed to the methyl and methylene protons which underwent deuteration, and observed that the corresponding signals due to the complexed ones become weaker and overlapped, as can be seen in Fig. S6 in Supplementary information. Thus, it was not possible to assign the signals due to the complexed protons and to estimate separately the deuteration rates catalyzed by primary and secondary hydroxy groups for the methyl and methylene protons. However, it is likely that the secondary hydroxy side of α-CD is more effective for the deuteration because of its stronger basicity and the larger number of hydroxy groups.

## Discussion

**Figure 5:**
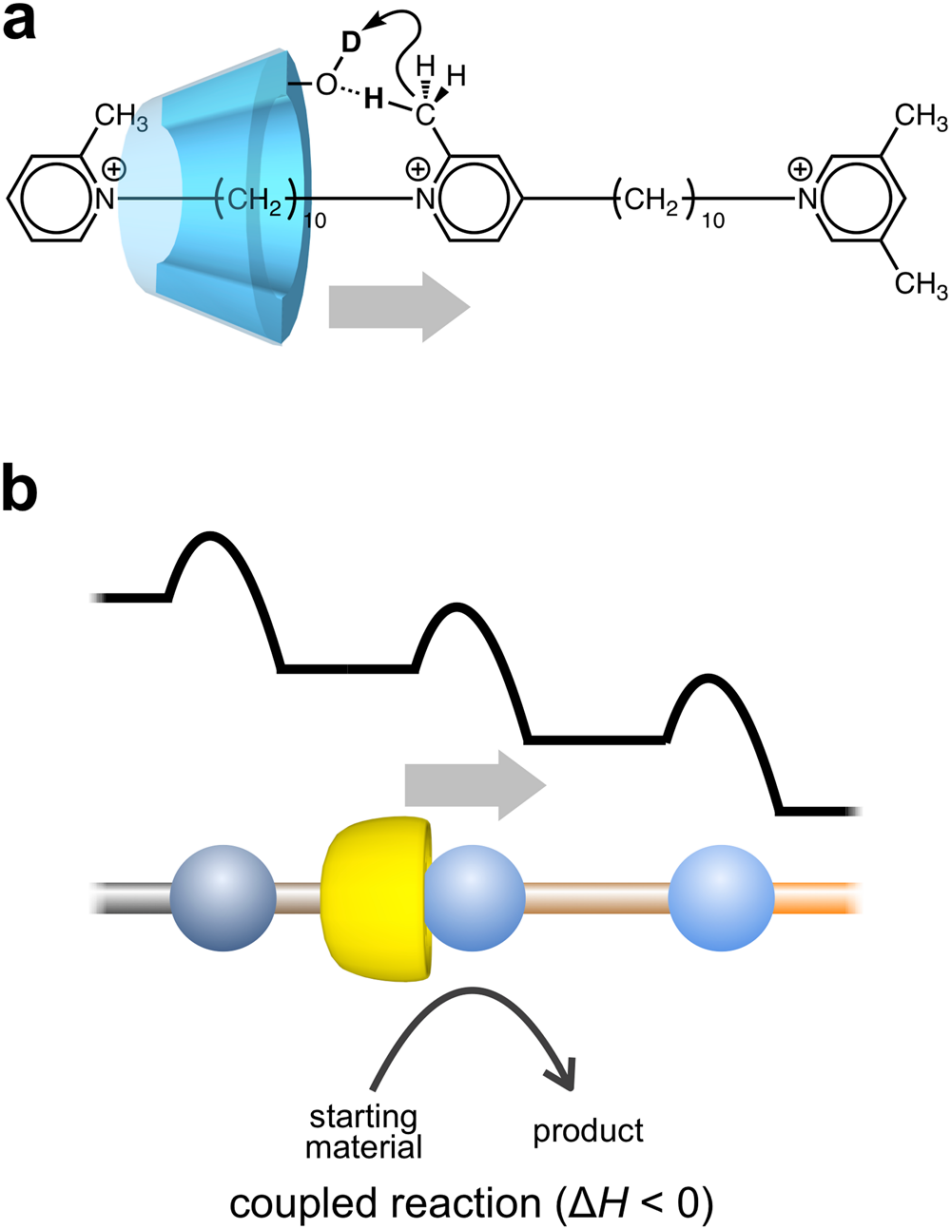
Conceptual illustration for translation of the rotor coupled with a reaction in (pseudo-)rotaxane. (**a**) Face-selective translation of α-CD coupled with deuteration in the pseudo-rotaxane in this study. (**b**) A ratchet-like translation in a (pseudo-)rotaxane, in which the translation of the rotor is coupled with a reaction driven enthalpically.

The data described in the former subsections have indicated that the face-selective translation of α-CD and the α-CD-catalyzed deuteration occur simultaneously in the pseudo-rotaxane. Here we discuss their relationship. The rate constants, *k*_−1_′, *k*_−1_″, *k*_2_′, *k*_2_″, *k*_−2_′, and *k*_−2_″, range 10^−15^–10^−9^ s^−1^, whereas *k*_app_ ranges 10^−15^–10^−11^ s^–1^. These data indicate that the time scales of translation and deuteration significantly overlap. On the other hand, the Arrhenius plot for *k*_app_ (Fig. 3d) shows a trapezoid shape. Since the deuteration occurs only in the complexed state of α-CD and the two-station axis molecule, it is likely that *k*_app_ is written as *k*_app_′*c*, where *c* can be assumed to be the molality of complexed axis molecule (*c* = [10] + [20] + [01] + [02] + [11] + [21] + [12] + [22]). Using the eight rate constants evaluated, the molalities of all the nine states of the two-station axis molecule can be calculated. If we choose the average molality of complexed axis molecule in the period of 0–5 days as a representative value for *c*, we can roughly estimate *k*_app_′ values at 50, 60, 70, 80, and 90 °C. The Arrhenius plot for *k*_app_′ indicates the slope is almost zero in the temperature range of 60–90 °C, indicative of the apparent activation energy of almost zero for the deuteration (Fig. S7 in Supplementary information). On the basis of these considerations, it is likely that the translation of α-CD and the α-CD-catalyzed deuteration are somehow coupled (Fig. 5a). Construction of a translational molecular ratchet, which regulates restrictedly the direction of movement, may require a large energy gradient with appropriate energy barriers and an enthalpically-driven coupled reaction (Fig. 5b). This work will provide a significant insight for construction of artificial molecular ratchet, which can be applied to artificial molecular motors with a high efficiency of energy conversion, comparable to biological ones.

## Methods

### Measurements

^1^H NMR spectra were obtained on a JEOL JNM ECA500 spectrometer or an Agilent INOVA 600 spectrometer. The ^1^H NMR signal of solvent was used as internal standard (2.49 ppm for DMSO-*d*_6_, at 30 °C and 4.79, 4.49, 4.40, 4.30, 4.21, and 4.13 ppm for D_2_O at 30, 50, 60, 70, 80, and 90 °C, respectively). An Agilent INOVA 600 spectrometer was also employed to obtain spectra of two dimensional J-correlation spectroscopy with gradient coherence selection (gCOSY), two dimensional total J-correlation spectroscopy for scalar coupled spin systems with a zero-quantum filter for artifact suppression (zTOCSY), and two dimensional heteronuclear single-quantum 1-bond J-correlation spectroscopy with adiabatic 180° X-nuclei pulses and gradient coherence selection (gHSQCAD). Deconvolution of ^1^H NMR spectra was carried out using a MestReNova software (version 10.0.2). Electrospray ionization mass spectroscopy (ESI-MS) data were recorded in a positive mode on a Thermo Fisher Scientific LTQ-Orbitrap-XL, controlled by the XCARIBUR 2.1 software package. The condition of ionization was set to the following parameters; ion spray voltage at 3.5 kV, ion spray temperature at 100 °C, and ion transfer tube temperature at 275 °C. Internal calibration of ESI-MS was carried out using the monoisotopic peaks of sodium adducted ion of diethylphthalate (*m*/*z* 314.1410), protonated ion of di-2-ethylhexylphthalate (*m*/*z* 391.2843), and sodium adducted ion of di-2-ethylhexyl-phthalate (*m*/*z* 413.2662).

### Density functional theory calculations

To investigate the structures of metastable complexed states of α-CD and the two-station axis molecule, DFT calculations were carried out for model systems composed of α-CD and a station model (i.e., *N*-decyl-2-methylpyridinium or 4-decyl-2,*N*-dimethylpyridinium) using the Gaussian 09 program^32^. In all the calculations, DFT with PBE0 functional was used, and 6–31 + G(d) basis sets were applied for the hydrogen, carbon, nitrogen, and oxygen atoms. All the geometries of the model systems were fully optimized.

## Electronic supplementary material

Supplementary information
