# Supplementary material for: Toward a translational molecular ratchet: face-selective translation coincident with deuteration in a pseudo-rotaxane
Source: Sci Rep. 2018 Jun 12;8:8950. doi: 10.1038/s41598-018-27226-2 (PMC5997654; doi:10.1038/s41598-018-27226-2)
Supplement: Supplementary file 1 — Supplementary information [file 41598_2018_27226_MOESM1_ESM.docx]

Supplementary information for

**Toward a translational molecular ratchet: face-selective translation coincident with deuteration in a pseudo-rotaxane**

Akihito Hashidzume^1^, Akihiro Kuse^1^, Tomoya Oshikiri^2^, Seiji Adachi^1^, Mitsutaka Okumura^1^, Hiroyasu Yamaguchi^1^, & Akira Harada^1,3^

^1^Graduate School of Science, Osaka University, 1-1 Machikaneyama-cho, Toyonaka, Osaka 560-0043, Japan. ^2^Research Institute for Electronic Science, Hokkaido University, Kita 21-jo, Nishi 10-chome, Kita-ku, Sapporo, 001-0021, Japan. ^3^JST-ImPACT, Chiyoda-ku, Tokyo 100-8914, Japan. Correspondence and requests for materials should be addressed to A.Harada (email: harada@chem.sci.osaka-u.ac.jp)

**Materials.** α-CD was purchased from Junsei Chemical Co., Ltd. (Tokyo, Japan) and purified by recrystallization in water, followed by drying at 80 °C under vacuum. 2-Picoline, 1,10-diiododecane, and 3,5-lutidine were obtained from Tokyo Chemical Industry Co., Ltd. (Tokyo, Japan). 2,4-Lutidine, 1,9-dibromonooctane, and a solution of butyllithium in hexane (3.4 M) were purchased from Tokyo Chemical Industry Co., Ltd. and 2,4-lutidine and 1,9-dibromonooctane were purified by distillation under an argon atmosphere. Dimethylamine was obtained from Kanto Chemical Co., Inc. (Tokyo, Japan) and purified by distillation under an argon atmosphere. Methyl iodide, alumina, and ammonium chloride were purchased from Wako Pure Chemical Industries, Ltd. (Osaka, Japan). Acetone, acetonitrile, hexane, dichloromethane, and hydrochloric acid were purchased from Nacalai Tesque, Inc. (Kyoto, Japan). Tetrahydrofuran (THF), purchased from Kanto Chemical Co., Inc., was purified utilizing a Glass Contour solvent dispending system. Water was purified by using a Millipore Milli-Q system. Other reagents were reagent grade and used without further purification.

PM-α-CD was prepared from α-CD with methyl iodide in the presence of sodium hydride using *N*,*N*-dimethylformamide as solvent according the procedure by Szejtli et al.^S1^.

**Preparation of the two-station axis molecule.** The two-station axis molecule was prepared according to Scheme S1^S2,S3^.

Scheme S1

Under an argon atmosphere, 2,4-lutidine (10.0 mL, 87 mmol) was dissolved in THF (180 mL) with a 250 mL Schlenk flask. The Schlenk flask was cooled down to –78 °C with a dry ice/methanol cooling bath. A solution of butyllithium in hexane (3.4 M, 50.0 mL, 170 mmol) was added to the solution by a syringe under an argon atmosphere. After the solution was stirred for 30 min, diethylamine (10.0 mL, 96 mmol) was added to the solution by a syringe under an argon atmosphere. After the solution was stirred for 30 min, the solution was added to a solution of 1,9-dibromononane (80.0 mL, 390 mmol) in THF (20 mL) by a syringe under an argon atmosphere. The reaction mixture was gradually warmed to room temperature, and stirred for 7 h. The solution was poured into an aqueous solution of ammonium chloride (0.5 M, 100 mL) to quench the reaction. After evaporation of the organic solvent at 0 °C, the residual mixture was dropped into 1 M hydrobromic acid (500 mL), and the product was extracted with dichloromethane (6 × 100 mL). The organic layers were combined, and dried with sodium sulfate (70 g). The organic layer was concentrated under reduced pressure. The product **1** was purified by reprecipitation with hexane (900 mL). The product **1** was recovered by filtration as a pale yellow powder (yield 28.3 g, 82 %). ^1^H NMR (500 MHz, DMSO-*d*_6_): *δ* 8.64 (d, *J* = 6.2 Hz, 1H), 7.78 (s, 1H), 7.72 (d, *J* = 6.0 Hz, 1H), 3.50 (t, *J* = 6.7 Hz, 2H), 2.79 (t, *J* = 7.6 Hz, 2H), 2.65 (s, 3H), 1.79-1.73 (m, 2H), 1.65-1.60 (m, 2H), 1.38-1.21 (m, 12H). Anal. Calcd for (C_16_H_27_NBr_2_)_1_(H_2_O)_0.5_: C, 47.78; H, 7.02; N, 3.48. Found: C, 47.70; H, 6.89; N, 3.32.

The obtained **1** (5.46 g, 13.8 mmol) was dissolved in water (30 mL). The aqueous solution was poured into a 1 M aqueous solution of sodium hydroxide (100 mL). The aqueous solution was extracted with dichloromethane (3 × 50 mL). The organic layer were combined, and dried with sodium sulfate (10 g). After evaporation of the solvent, the product **2** was obtained in a quantitative yield. ^1^H NMR (300 MHz, DMSO-*d*_6_): *δ* 8.28 (d, *J* = 5.1 Hz, 1H), 7.05 (s, 1H), 6.98 (d, *J* = 4.0 Hz, 1H), 3.50 (t, *J* = 6.8 Hz, 2H), 2.51 (t, *J* = 7.7 Hz, 2H), 2.40 (s, 3H), 1.80-1.74 (m, 2H), 1.58-1.51 (m, 2H), 1.38-1.20 (m, 12H).

3,5-Lutidine was pretreated with activated alumina. The pretreated 3,5-lutidine (3.0 mL, 26 mmol) and **2** (4.31 g, 14 mmol) were dissolved in acetone (5 mL) using a 50 mL flask equipped with a condenser. The solution was heated under reflux overnight with an oil bath thermostated at 60 °C. 3,5-Lutidine (1.0 mL, 8.7 mmol) was added to the solution. The solution was heated under reflux for 2 days with an oil bath thermostated at 60 °C. When the solution was poured into diethyl ether (100 mL), the crude product was obtained as a brown oil. The brown oil was washed with diethyl ether (3 × 50 mL), and then dissolved in water (20 mL). After the aqueous solution was poured into 1 M sodium hydroxide (75 mL), the product was extracted with dichloromethane (3 × 50 mL). The organic layer was collected and dried with anhydrous sodium sulfate (10 g). After evaporation of the solvent, the product **3** was obtained as dark green oil (yield 3.52 g, 61 %). ^1^H NMR (500 MHz, DMSO-*d*_6_): *δ* 8.78 (s, 2H), 8.28 (d, *J* = 3.05 Hz, 1H), 8.27 (s, 1H), 7.05 (s, 1H), 6.98 (d, *J* = 5.1 Hz, 1H), 4.44 (t, *J* = 7.4 Hz, 2H), 2.52-2.49 (m, 2H), 2.43 (s, 6H), 2.39 (s, 3H), 1.90-1.85 (m, 2H), 1.56-1.50 (m, 2H), 1.36-1.19 (m, 12H).

2-Picoline (0.30 mL, 3.1 mmol) and 1,10-diiododecane (13.0 g, 33 mmol) were dissolved in acetone (20 mL) with a 50 mL flask equipped with a condenser. The solution was heated under reflux using an oil bath thermostated at 60 °C for 4 days. The product was purified by reprecipitation with hexane (300 mL), and then recovered by filtration. After drying, the product **4** was obtained as yellow powder (yield 1.30 g, 87 %). ^1^H NMR (500 MHz, DMSO-*d*_6_): *δ* 8.96 (d, *J* = 5.1 Hz, 1H), 8.44 (t, *J* = 7.8 Hz, 1H), 8.02 (d, *J* = 7.9 Hz, 1H), 7.94 (t, *J* = 6.8 Hz,1H), 4.51 (t, *J* = 7.8 Hz, 2H), 3.25 (t, *J* = 6.8 Hz, 2H), 2.82 (s, 3H), 1.85-1.79 (m, 2H), 1.75-1.69 (m, 2H), 1.36-1.22 (m, 12H).

The obtained **3** (1.65 g, 3.9 mmol) and **4** (1.14 g, 2.3 mmol) were dissolved in acetonitrile (20 mL) with a test tube (Φ 30 mm, height 190 mm). Alumina (1.5 g) was added to the solution. After a condenser was equipped with the flask, the solution was heated under reflux for 8 days with a chambers thermostated at 70 °C. The residual solution was dissolved in water (50 mL), and then washed with dichloromethane (5 × 100 mL). After evaporation of the solvent under reduced pressure, the desired two-station axis molecule was obtained as a brown oil (yield 0.612 g, 22 %). ^1^H NMR (500 MHz, DMSO-*d*_6_): *δ* 8.99 (d, *J* = 5.2 Hz, 1H), 8.85 (d, 6.5 Hz, 1H), 8.83 (s, 2H), 8.46 (t, *J* = 7.8 Hz, 1H), 8.29 (s, 1H), 8.04 (d, *J* = 8.3 Hz, 1H), 7.96 (t, *J* = 6.5 Hz, 1H), 7.91 (s, 1H), 7.82 (d, *J* = 6.5 Hz, 1H), 4.52 (t, *J* = 7.9 Hz, 2H), 4.47 (t, *J* = 7.5 Hz, 2H), 4.45 (t, *J* = 8.2 Hz, 2H), 2.83 (s, 3H), 2.79 (t, *J* = 8.2 Hz, 2H), 2.77 (s, 3H), 2.45 (s, 6H), 1.93-1.86 (m, 2H), 1.84-1.77 (m, 4H), 1.66-1.60 (m, 2H), 1.37-1.21 (m, 24H).

The two-station axis molecule was characterized by various NMR techniques including gCOSY, zTOCSY, and gHSQCAD measurements. Fig. S1 demonstrates a typical example of ^1^H NMR spectra of the axis molecule as well as full assignments. The spectrum contains signals assignable to protons of the pyridinium residues in the region of 7.6 – 8.7 ppm, where “p*nx*” denotes a specific proton of the *n*-th pyridinium from the gate. Signals due to methylene protons directly attached to N atoms of the pyridinium are seen at ca. 4.5 ppm. Here “s*nx*” denotes specific methylene protons in the *n*-th station. There are signals ascribed to methyl and methylene protons bound to a pyridinium in the region of 2.5 – 2.9 ppm, where “m*nx*” denotes specific methyl protons on the *n*-th pyridinium. The spectrum also exhibits signals due to methylene protons in the stations in the region of 1 – 2 ppm. The characterization data indicate that the desired axis molecule was successfully prepared.

Figure S1

**A simplified kinetic model for the formation of pseudo-rotaxane.** When the molality of the *xy* state is expressed by [*xy*], the following differential equations are derived based on the simplified kinetic model shown in Fig. 2a.

 (1)

 (2)

 (3)

 (4)

 (5)

 (6)

 (7)

 (8)

 (9)

 (10)

From the mass conservation law,

 (11)

 (12)

Here, [00]_0_ and [α-CD]_0_ represent the initial concentrations of the two-station axis molecules and α-CD, i.e., the total concentrations, respectively. It is almost impossible to solve analytically these simultaneous differential equations. It is however possible to calculate the time evolution of molalities of all the states when values for the eight rate constants, i.e., *k*_1_*'*, *k*_–1_*'*, *k*_1_*"*, *k*_–1_*"*, *k*_2_*'*, *k*_–2_*'*, *k*_2_*"*, and *k*_–2_*"*, are given, because [00]_0_ and [α-CD]_0_ are known.

**References**

S1. Shapiro, I. O., Terekhova, M. I., Ranneva, Y. I., Petrov, E. S. & Shatenshtein, A. I. Broensted relation in carbon acidity of toluene and methyl derivatives of biphenyl, pyridine, and quinoline. *Zh. Obshch. Khim.* **53**, 1386-1391 (1983).

S2. Oshikiri, T., Yamaguchi, H., Takashima, Y. & Harada, A. Face selective translation of a cyclodextrin ring along an axle. *Chem. Commun.*, 5515-5517, doi:10.1039/b906425g (2009).

S3. Hashidzume, A. *et al.* A pseudo-rotaxane of α-cyclodextrin and a two-station axis molecule consisting of pyridinium and decamethylene moieties, and its deuteration in deuterium oxide. *Tetrahedron* **73**, 4988-4993, doi:10.1016/j.tet.2017.05.044 (2017).

S4. Oshikiri, T., Takashima, Y., Yamaguchi, H. & Harada, A. Kinetic Control of Threading of Cyclodextrins onto Axle Molecules. *J. Am. Chem. Soc.* **127**, 12186-12187, doi:10.1021/ja053532u (2005).


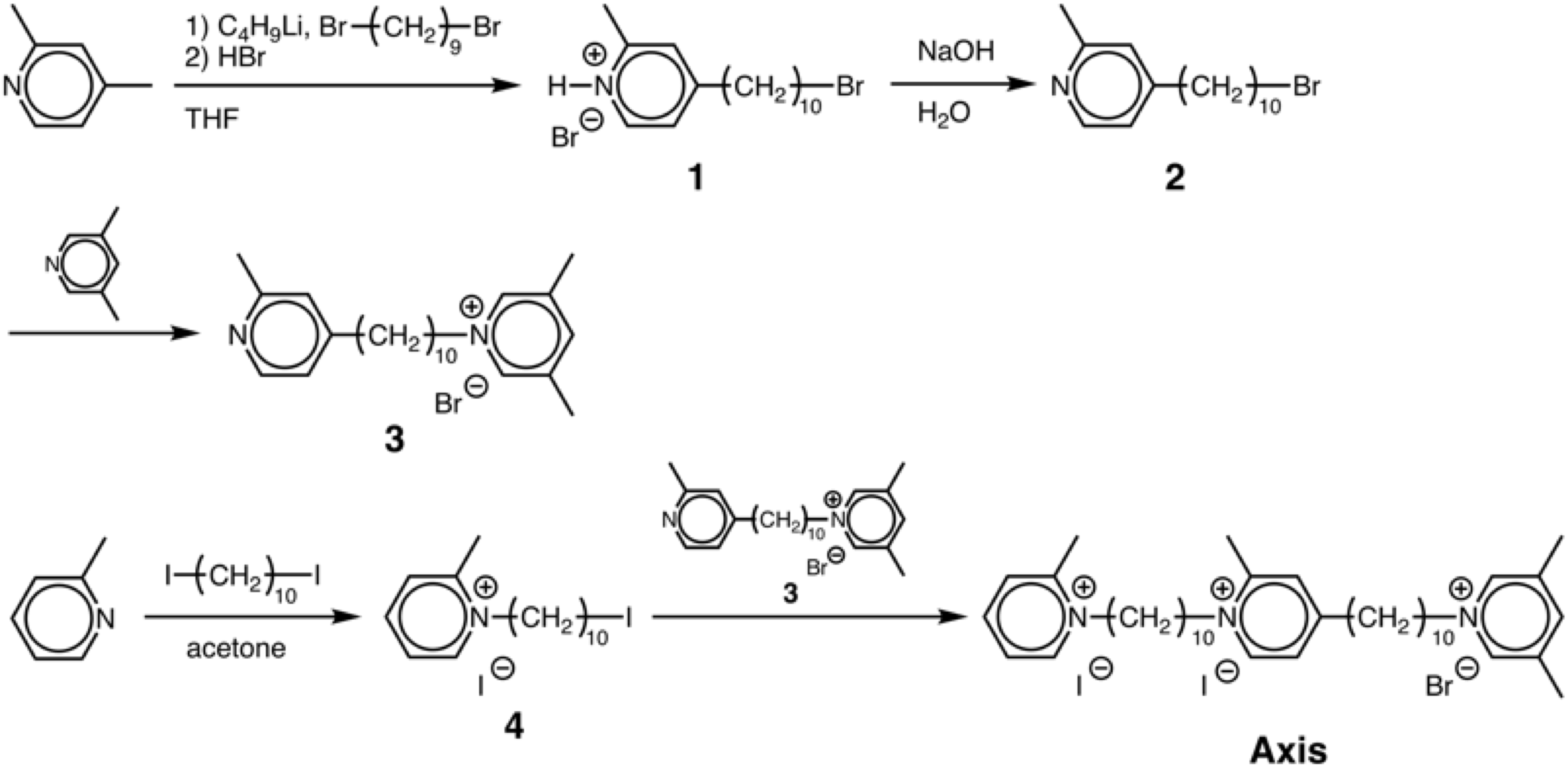


**Scheme S1. Synthesis of the two-station axis used in this study.** The two-station axis molecule was prepared step by step in a manner similar to our previous reports ^28,29^. 2,4-Lutidine was lithiated with butyllithium and coupled with 1,9-dibromononane to form **1**. After **1** was treated with sodium hydroxide, **2** was obtained. The second station part, **3**, was prepared by reaction of **2** with 3,5-lutidine. 2-Picoline was mixed with 1,10-diiododecane to yield **4**. Coupling of **3** with **4** gave the desired two-station axis molecule.


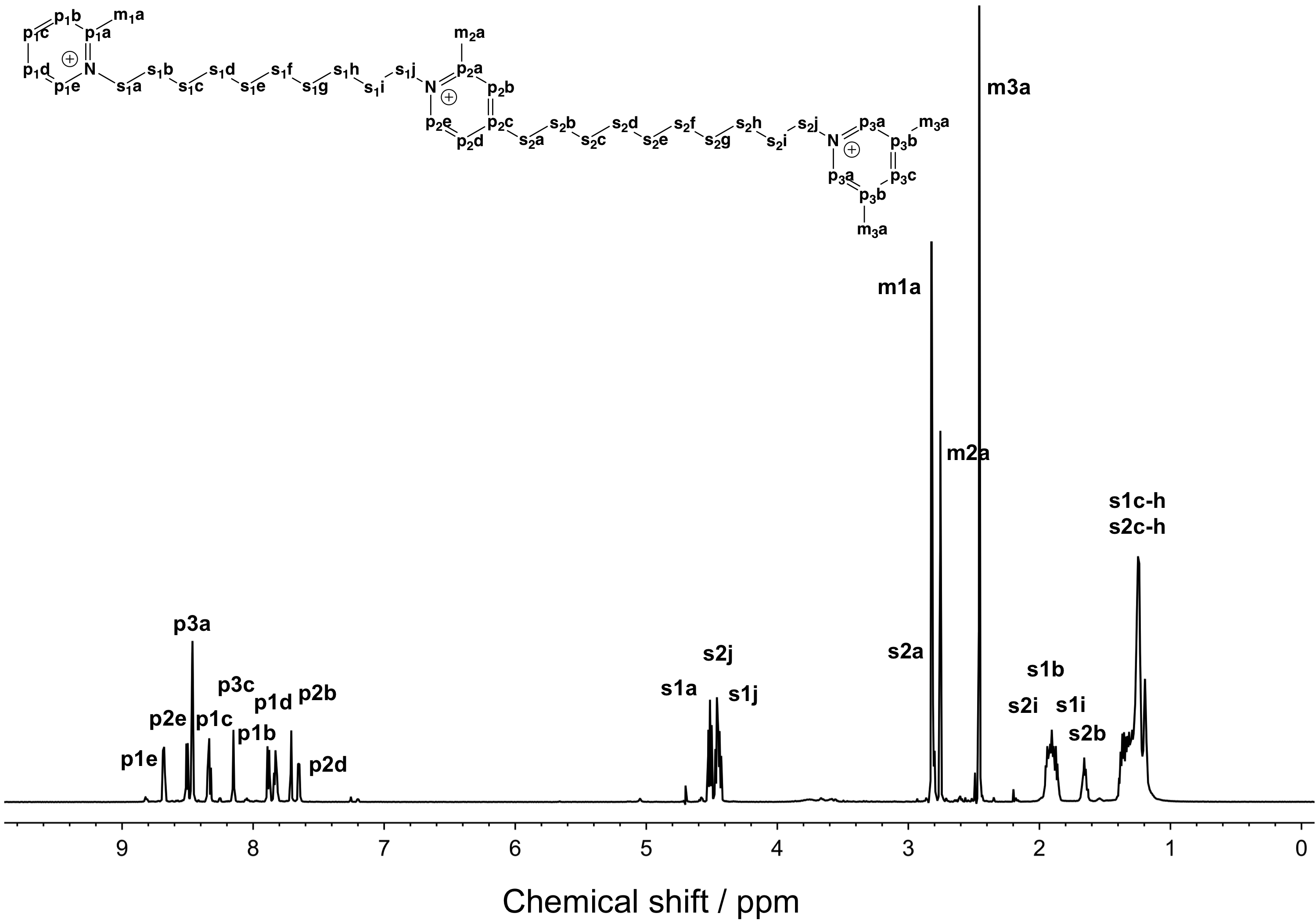


**Figure S1. ^1^H NMR spectrum and assignments for the two-station axis molecule at 30 °C in D_2_O.** The spectrum contains signals assignable to protons of the pyridinium residues in the region of 7.6 – 8.7 ppm, where “p*nx*” denotes a specific proton of the *n*-th pyridinium from the gate. Signals due to methylene protons directly attached to N atoms of the pyridinium are seen at ca. 4.5 ppm. Here “s*nx*” denotes specific methylene protons in the *n*-th station. There are signals ascribed to methyl and methylene protons bound to a pyridinium in the region of 2.5 – 2.9 ppm, where “m*nx*” denotes specific methyl protons on the *n*-th pyridinium. The spectrum also exhibits signals due to methylene protons in the stations in the region of 1 – 2 ppm.


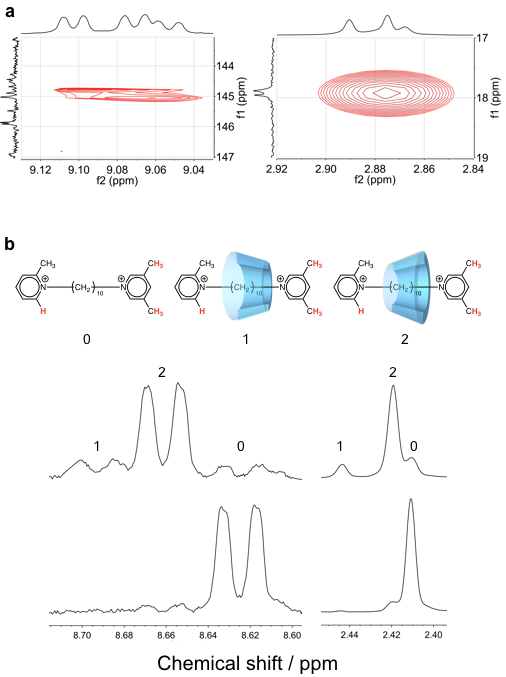


**Figure S2. Assignment of ^1^H NMR signals of the pseudo-rotaxane formed from the two-station axis molecule and α-CD in D_2_O.** **(a)** HSQC (gHSQCAD) spectra for the binary mixture of the two-station axis molecule and α-CD in D_2_O at 70 °C. **(b)** ^1^H NMR spectra for the proton at 6-position of 2-methylpyridinium (left) and the methyl protons of 3,5-dimethylpyridnium (right) in a binary mixture of a one-station axis molecule and α-CD heated at 70 °C after 1 (lower) and 63 days (upper)^S4^.


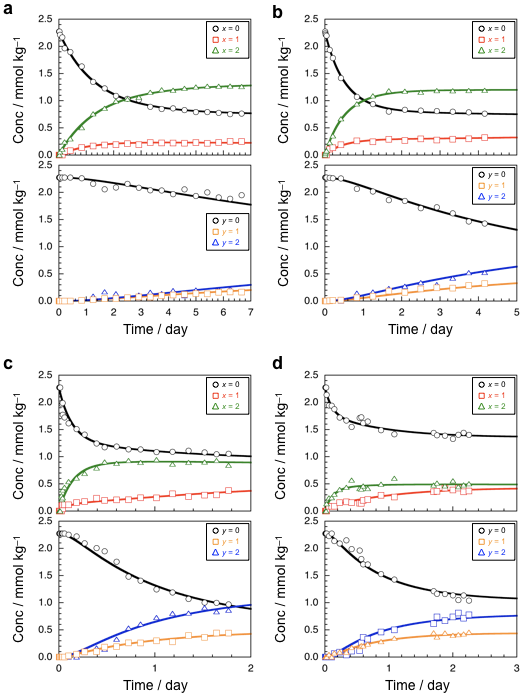


**Figure S3. Time evolution of the concentrations of free and complexed stations of the two-station axis molecule in D_2_O; the first (upper) and second stations (lower), and free (circle) and included by α-CD from the primary (square) and secondary sides (triangle), respectively.** **(a)** At 50 °C. **(b)** At 60 °C. **(c)** At 80 °C. **(d)** At 90 °C.


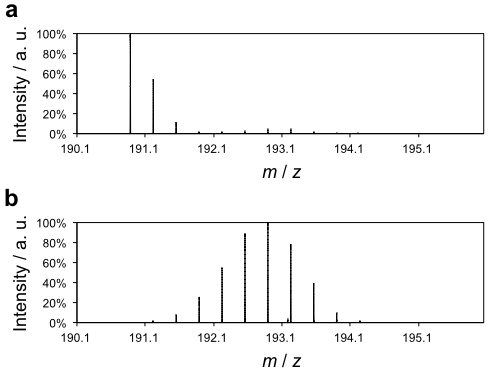


**Figure S4. Mass spectra of the two-station axis molecule.** **(a)** The MS chart for the axis molecule just dissolved in H_2_O at room temperature, which contains the trivalent molecular ion peak at *m*/*z* = 190.83 (i.e., *m* = 572.49). **(b)** The MS chart for the axis molecule heated in D_2_O at 70 °C for 2 days in the presence of α-CD, which exhibits a series of signals centered at *m*/*z* = 192.84, which is assignable to a trivalent molecular ion peak of *m* = 578.52.


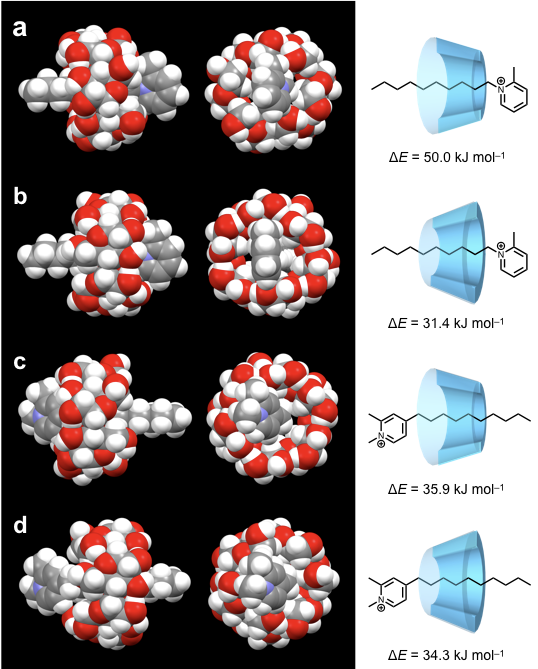


**Figure S5. Side and top views of fully optimized structures for the model systems composed of α-CD and a station model.** **(a)** and **(b)** The first station model included by α-CD from the primary **(a)** and secondary hydroxy sides **(b)**, respectively. **(c)** and **(d)** The second station model included by α-CD from the primary **(c)** and secondary hydroxy sides **(d)**, respectively.


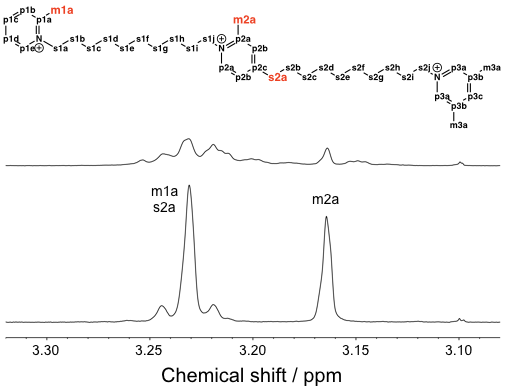


**Figure S6. ^1^H NMR spectra for a binary mixture containing the two-station axis molecule (2.3 mmol kg^–1^) and α-CD (9.1 mmol kg^–1^) in D_2_O at 70 °C just after heating (lower) and after heating for 0.83 day (upper).**


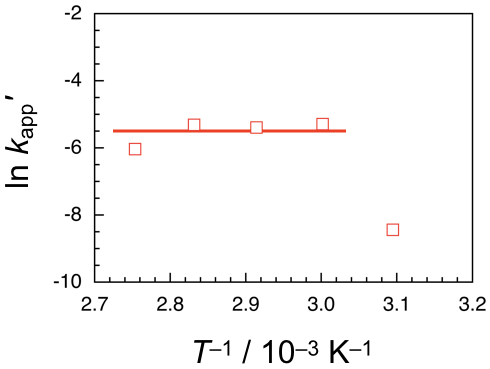


**Figure S7. Arrhenius plot for *k*_app_*'* (= *k*_app_/*c*, where *c* denotes the molality of complexed axis molecule after 1 day).** The slope of this plot is almost zero in the temperature range of 60 – 90 °C, indicating that the apparent activation energy of deuteration is ca. 0.

**Table S1. Apparent rate constants for the formation of pseudo-rotaxane.**

| temp. / °C | *k*_1_*'* / s^–1^ mol^–1^ kg | *k*_–1_*'* / s^–1^ | *k*_1_*"* / s^–1^ mol^–1^ kg | *k*_–1_*"* / s^–1^ | *k*_2_*'* / s^–1^ | *k*_–2_*'* / s^–1^ | *k*_2_*"* / s^–1^ | *k*_–2_*"* / s^–1^ |
| --- | --- | --- | --- | --- | --- | --- | --- | --- |
| 50 | 1.3 × 10^–4^ | 2.0 × 10^–6^ | 5.2 × 10^–4^ | 1.7 × 10^–6^ | 2.0 × 10^–6^ | 5.0 × 10^–7^ | 5.8 × 10^–7^ | 6.7 × 10^–7^ |
| 60 | 3.5 × 10^–4^ | 3.3 × 10^–6^ | 1.3 × 10^–3^ | 4.5 × 10^–6^ | 4.2 × 10^–6^ | 1.7 × 10^–6^ | 2.0 × 10^–6^ | 1.7 × 10^–6^ |
| 70 | 5.0 × 10^–4^ | 3.2 × 10^–6^ | 2.7 × 10^–3^ | 1.7 × 10^–5^ | 2.5 × 10^–5^ | 1.7 × 10^–5^ | 6.0 × 10^–6^ | 3.3 × 10^–6^ |
| 80 | 8.0 × 10^–4^ | 9.2 × 10^–6^ | 3.5 × 10^–3^ | 2.3 × 10^–5^ | 5.0 × 10^–5^ | 3.3 × 10^–5^ | 1.4 × 10^–5^ | 6.7 × 10^–6^ |
| 90 | 7.5 × 10^–4^ | 1.7 × 10^–5^ | 2.0 × 10^–3^ | 3.8 × 10^–5^ | 6.7 × 10^–5^ | 2.0 × 10^–5^ | 2.5 × 10^–5^ | 1.2 × 10^–5^ |

**Table S2. Apparent frequency factors (*A*_app_) and activation energies (*E*_app_) for the formation of pseudo-rotaxane.**

|  | *A*_aap_ | *E*_app_ / kJ mol^–1^ |
| --- | --- | --- |
| *k*_1_*'* | 1.2 × 10^3^ | 42 ± 9 |
| *k*_–1_*'* | 2.8 × 10^2^ | 51 ± 10 |
| *k*_1_*"* | 7.3 × 10^2^ | 37 ± 16 |
| *k*_–1_*"* | 7.3 × 10^6^ | 78 ± 9 |
| *k*_2_*'* | 2.4 × 10^9^ | 93 ± 12 |
| *k*_–2_*'* | 1.2 × 10^13^ | 120 ± 13 |
| *k*_2_*"* | 6.8 × 10^8^ | 93 ± 6 |
| *k*_–2_*"* | 1.2 × 10^5^ | 70 ± 2 |

**Table S3. Apparent rate constants (*k*_app_) for the deuteration of the axis molecule in D_2_O at 70 °C in the absence and presence of α-CD, β-CD, γ-CD, PM-α-CD, dextrin, and MGlc.**

| additive | *k*_app_ / s^–1^ |
| --- | --- |
| – | (2.4 ± 0.8) ×10^–7^ |
| α-CD | (7.7 ± 0.2) × 10^–6^ |
| β-CD | (3.1 ± 0.1) ×10^–6^ |
| γ-CD | (4.2 ± 0.5) ×10^–7^ |
| PM-α-CD | (5.4 ± 0.1) ×10^–8^ |
| dextrin | (1 ± 0.2) ×10^–7^ |
| MGlc | (3.0 ± 0.4) ×10^–7^ |

**Table S4. Apparent rate constants (*k*_app_) for the deuteration of the axis molecule in D_2_O at different temperatures in the presence of α-CD.**

| temperature / °C | *k*_app_ / s^–1^ |
| --- | --- |
| 50 | (2.6 ± 0.1) ×10^–7^ |
| 60 | (8.0 ± 0.2) × 10^–6^ |
| 70 | (7.7 ± 0.2) ×10^–6^ |
| 80 | (8.9 ± 0.2) ×10^–6^ |
| 90 | (3.5 ± 0.3) ×10^–6^ |
